# INVESTIGATING THE CONNECTION BETWEEN AGEISM AND ELDER ABUSE

**DOI:** 10.1093/geroni/igac059.2287

**Published:** 2022-12-20

**Authors:** David Burnes, Karl Pillemer, Andie MacNeil

**Affiliations:** University of Toronto, Toronto, Ontario, Canada; Cornell University, Ithaca, New York, United States; University of Toronto, Toronto, Ontario, Canada

## Abstract

Elder abuse is recognized as a pervasive public health problem with detrimental consequences for older adults and society. Although considerable research has examined elder abuse risk factors at the individual level, there is a growing call for the field to move beyond proximal causes and consider broader socio-cultural and structural factors that influence elder abuse. Illustrating this shift, organizations, advocacy groups and researchers have proposed a connection between ageism and elder abuse. However, despite the assertion that ageism is a causal factor for elder abuse, there is a scarcity of research to demonstrate this relationship, and a coherent theoretical framework linking ageism to elder abuse remains to be articulated. The purpose of the current study was to examine the conceptual pathways and limited empirical research connecting ageism and elder abuse, and to develop a conceptual model that links ageism and elder abuse. We conducted a comprehensive review and synthesis of the ageism/elder abuse literature, as well as research from other domains of interpersonal/family violence. Based on this synthesis, the proposed model includes plausible mediators (social isolation, devaluation, depersonalization, infantilization, powerlessness, blame) and moderators (intersection with socio-cultural identities, internalized ageism, policy/social norms) that could be targeted as mechanisms of change in interventions designed to address the issue. As such, it provides a framework for hypothesis-testing and future research on the topic. This study informs a research agenda to bring conceptual clarity and empirical evidence to the study of the connection between ageism and elder abuse.

